# Possible Roles of mmu-miR-141 in the Endometrium of Mice in Early Pregnancy Following Embryo Implantation

**DOI:** 10.1371/journal.pone.0067382

**Published:** 2013-06-25

**Authors:** Xueqing Liu, Rufei Gao, Xuemei Chen, Hailing Zhang, Anshun Zheng, Dehui Yang, Yubin Ding, Yingxiong Wang, Junlin He

**Affiliations:** Laboratory of Reproductive Biology, School of Public Health, Chongqing Medical University, Yuzhong District, Chongqing, PR China; State Key Laboratory of Reproductive Biology, Institute of Zoology, Chinese Academy of Sciences, China

## Abstract

**Objective:**

Embryo implantation is directly affected by genes related to uterine receptivity. Studies have demonstrated the important roles of miRNAs in the regulation of gene expression. Our early miRNA chip analyses revealed that the mmu-miR-141 expression in endometrial tissue is lower after embryo implantation than before it. However, the possible roles of miR-141 in embryo implantation have not yet been elucidated. Here, mmu-miR-141 was designed to detect the expression and role of miR-141 in the endometria of mice in early pregnancy following embryo implantation.

**Methods:**

Real-time PCR and in-situ hybridization were used to study mmu-miR-141 expression in mouse uterus. Cell proliferation was detected by tetrazolium dye (MTT) assay and flow cytometry. Real-time PCR and Western blot analysis were used to confirm the mRNA and protein levels of phosphatase and tensin homolog (PTEN) to determine whether it was the target gene of mmu-miR-141. Enhanced green fluorescent protein (EGFP) fluorescence reporter vector analysis was also performed. A functional study was performed by injecting mice uteri with mmu-miR-141 inhibitor or mimic vectors.

**Results:**

mmu-miR-141 expression was lower on day 6 (D6) than day 4 (D4) and could be increased by progesterone. Reduced mmu-miR-141 could decrease the proliferation activity of stromal cells and promote apoptosis. Upregulation of mmu-miR-141 inhibited PTEN protein expression but downregulation of mmu-miR-141 increased it, while the mRNA level remained unchanged. EGFP fluorescence reporter vector analysis showed that miR-141 targets the 3′-untranslated region of the PTEN mRNA. In addition, when the physiological mmu-miR-141 level was altered on D2 by injecting with inhibitor or mimic, the embryo implantation sites were significantly decreased on D7.

**Conclusions:**

This study demonstrated that mmu-miR-141 might influence cell proliferation and apoptosis in the endometrium by negatively regulating PTEN expression, and could also influence the number of embryo implantation sites. mmu-miR-141 plays an essential role in embryo implantation.

## Introduction

MicroRNAs (miRNAs) are a growing class of endogenous, small, noncoding RNAs that regulate gene expression at the post-transcriptional level by binding to the 3′-untranslated region (UTR) of its target gene mRNA for translational repression, degradation, or both [1], [2]. This gene regulation by miRNA depends on sequence complementarities between the miRNA and its target miRNA responsive element (MRE) and on the total number of MREs in a given 3′-UTR [3], [4]. Evidence from many studies suggests that miRNAs regulate tissue-specific differentiation and development [5] and play essential functions in multiple biological pathways and diseases, ranging from embryo development, cell fate determination, and apoptosis to immune response [6]–[10].

Embryo implantation is a complex reproductive process. Successful embryo implantation depends on the synchronized reciprocal interaction between blastocysts and uterus. This process is established and maintained by a series of cytokines that are involved in physiological changes of the endometrium. Embryo implantation is directly affected by abnormal expression of the genes related to the establishment of uterine receptivity, leading to spontaneous abortion [11]–[13]. MiRNAs are known to play an important function in the precise regulation of gene expression. A series of studies has shown that miRNAs play an essential role not only in the pathology but also in the physiology, including embryo implantation. In a study by Hu et al., a miRNA chip was used to examine the differential expression of miRNAs in the mouse uterus between implantation sites and within implantation sites. They found that 13 miRNAs were upregulated by at least 2-fold and two miRNAs were downregulated by at least 2-fold across different implantation sites [14]. Chakrabarty et al. found that mmu-miR-101a and mmu-miR-199a* were spatiotemporally expressed in the mouse uterus during implantation concurrently with the expression of the cyclooxygenase-2 gene, which is critical for embryo implantation [4]. Revel et al. demonstrated the role played by miRNAs in human embryo implantation defects [15]. Recently, Altmäe and colleagues reported that miR-30b, miR-30d, and miR-494 play important roles in human endometrial receptivity [16]. Together, these results indicate the importance of miRNAs in embryo implantation.

In our previous study [17], we used miRNA chip technology to study miRNA expression before and after embryo implantation. The results showed that the mmu-miR-141 expression in endometria after implantation (D6) was lower than that before implantation (D4). Furthermore, recent studies demonstrated that reduction in the miR-141 expression level is induced by leukemia inhibitory factor, which was then found to inhibit proliferation in the choriocarcinoma cell line JEG-3 [18]. However, the possible roles of miR-141 in embryo implantation are not yet known. Here, we aimed to detect the expression and roles of mmu-miR-141 in the endometrium of mice during embryo implantation. The findings of this study will provide an experimental basis for further understanding the molecular mechanism of embryo implantation.

## Materials and Methods

### Ethics Statement

All animal procedures were approved by the Ethics Committee of Chongqing Medical University.

### Animals

Six- to eight-week-old female NIH mice (weight range: 25–30 g) were provided for experimental use by the Laboratory Animal Center of Chongqing Medical University [Chongqing, China, Certificate: SCXK(YU) 20050002]. The animals were caged in a specific pathogen-free animal room under a controlled environment (14∶10 h light/dark photoperiod). The mice were mated with fertile male NIH mice at a ratio of 2∶1 (day of vaginal plug = D1). The pregnant mice were randomly divided into two groups each containing 20 mice. The mice in one group were sacrificed at 1000h on D4 and the other, at 1000h on D6. The endometria were separated from myometria and immediately stored in liquid nitrogen for analysis.

### Steroid Hormone Treatment Models

Normal sexually mature virginal mice were ovariectomized. 14 days after ovariectomy operation, the ovariectomized mice were randomly divided into four groups each containing 6 mice. Ovariectomized mice were treated with estradiol-17β 100 ng/mouse (E2 group), progesterone 2 mg/mouse (P group), or a combination of estradiol-17β and progesterone (E2+P group). Controls received vehicle only (Control group). All steroids were dissolved in sesame oil and injected subcutaneously. Mice uteri were collected 24 h after injection and frozen in liquid nitrogen for analysis.

### Real-time PCR

Total RNA was extracted from 50–100 mg endometrial tissue using Trizol reagent (Invitrogen, USA) according to the manufacturer’s instructions. The extracted RNA was quantified and assessed for purity by spectrometry at 260 and 280 nm, respectively. RNA integrity was examined by agarose gel electrophoresis. cDNA was synthesized with 0.5 µg total RNA in a 10-µL reaction system using First-Strand Synthesis System for RT-PCR (TaKaRa, China). Transcripts were quantified using SYBR Premix Ex Taq kits (TaKaRa) in a Light Cycler (iQ5 Muliticolor Real-Time PCR Detection System, BioRad). The real-time PCR master mixture (25 µL) contained 12.5 µL of 2× SYBR Premix Ex Taq, 1 µL of 10 pmol/µL primers, 2 µL of cDNA, and 8.5 µL of double-distilled H_2_O. The PCR conditions were as follows: initial denaturation at 95°C for 30 s; 40 cycles of 5 s at 95°C (denaturation) and 30 s at 60°C; followed by 72°C for 5 s. Experiments were performed in triplicate for each sample. The primers used in this study are listed in Table 1. U6 and β-actin were used to normalize the miR-141 and phosphatase and tensin homolog (PTEN) levels, respectively. The relative gene expression levels were calculated with the 2^−ΔΔCT^ method [19].

**Table 1:** Primers used for Real-time RT-PCR.

	Primers	Sequence (5′→3′)
miR-141	RT primer	GTCGTATCCAGTGCAGGGTCCGAGGTATTCGCACTGGATACGACCCTTCT
	PCR primer- Forward	CCGGGTAACACTGTCTGGTAAAG
	PCR primer- Reverse	GTGCAGGGTCCGAGGT
U6	RT primer	CGCTTCACGAATTTGCGTGTCAT
	PCR primer- Forward	GCTTCGGCAGCACATATACTAAAAT
	PCR primer- Reverse	CGCTTCACGAATTTGCGTGTCAT
PTEN	PCR primer- Forward	CCGTTACCTGTGTGTGGTGATATC
	PCR primer- Reverse	GAATGTATTTACCCAAAAGTGAAACATT
β-actin	PCR primer- Forward	TCGTGCGTGACATCAAAGAC
	PCR primer- Reverse	CAAGAAGGAA GGCTGGAAAA

### In-situ Hybridization

The mmu-miR-141-specific probe and negative control (Scramble) were purchased from Exiqon (Denmark). An in-situ hybridization kit (Beijing dinguo biotechnology company, China) was used for hybridization of the probe according to the manufacturer’s instructions. Frozen endometeria were sectioned and then fixed with 4% paraformaldehyde at room temperature for 10 min. The fixed samples were incubated with acetylate for 10 min, treated with protease K at 37°C for 5 min, after which they were incubated in prehybridization solution for 4 h at 50°C. Then, 40 µM/L of denatured probes was added to the samples and incubated overnight at 50°C. They were then washed with standard saline citrate (SSC) and incubated with BSA (1∶100 dilution) at 37°C for 1 h followed by incubation with AP labeled goat anti-rabbit IgG (1∶100 dilution) for 1 h. The samples were stained with NBT/BCIP at room temperature for 30 min.

### Cell Culture and Transfection

Mouse uterine stromal cells were isolated and purified, as previously described [20]. Pregnant mice were sacrificed at D4 and the uterus was removed. After removing the fat tissue and washing with phosphate-buffered saline (PBS), the uterus was cut into small pieces and treated with 1 mL 10% trypsin at 4°C for 1 h, 20°C for 1 h, and 37°C for 10 min. Digestion was terminated by adding DMEM/F12 containing 10% fetal bovine serum (FBS; Hangzhou Evergreen Biological Engineering Materials Co., Ltd.). The cells were collected by centrifugation at 500 rpm for 10 min, seeded in 50-ml culture flasks at a density of 1×10^6^ cells/mL, and cultured in DMEM/F12 medium (HyClone Laboratories, Inc., USA) supplemented with 10% FBS at 37°C in a 5% CO_2_ atmosphere. The medium was replaced every 4 h to remove nonadherent cells. Stromal cells were found to be intensively stained for vimentin. The cells used for transfection had a purity of more than 95%. Lipofectamine™ 2000 (lipo2000; Invitrogen) was mixed with the mmu-miR-141 mimic and mmu-miR-141 inhibitor (Guangzhou RiboBio Co., Ltd., China) and incubated for 20 min at room temperature, according to the manufacturer’s instructions. The cells were plated in 96-well plates at a density of 5,000 cells/well. miRNA mixtures were added into the cells for incubation at 37°C. Cells were harvested after culturing for 96 h.

### Flow Cytometry for Cell Cycle and Apoptosis Analysis

The stromal cells transfected by mmu-miR-141 inhibitor and negative controls were harvested. For apoptosis analysis, the samples were incubated with Annexin V and PI for 15 min in the dark at room temperature and quantitatively analyzed by a FACSVantage SE flow cytometer (BD Biosciences, USA). For cell cycle analysis, the cells were fixed with 75% ice-cold alcohol for more than 12 h. After the cells were washed twice, they were resuspended with PBS containing PI (1 mg/mL) and RNase (50 µg/mL) and incubated for 30 min at 37°C. The cell cycle was detected using FACSVantage SE flow cytometer. All experiments were performed in triplicate.

### MTT Assay

Cells transfected with different concentrations of mmu-miR-141 inhibitor (50, 100, and 150 nM) were incubated in 96-well plates for 3-(4,5-dimethyl-2-thiazolyl)-2,5-diphenyl 2H-tetrazolium bromide (MTT; Sigma, USA) assay. After 24, 48, and 72 h, 20 µL of 5 mg/mL MTT was added to each well. The cells were then incubated at 37°C for 4 h, after which the supernatants were removed carefully. Then, 150 µL of dimethyl sulfoxide (DMSO) was added to each well and thoroughly mixed for 10 min. The OD of each well was measured at 490 nm. All the experiments were performed in triplicate.

### Western Blotting

The mmu-miR-141 inhibitor- and mimic-transfected stromal cells (1×10^7^) were harvested for protein extraction using cell lysis buffer for Western blot analysis and immunoprecipitation (Beyotime, China). The protein concentration was determined using the BCA Protein Assay kit according to the manufacturer’s protocol (Beyotime). Proteins were separated and transferred to polyvinylidene difluoride membranes (BioRad, USA). The membranes were blocked with 5% nonfat milk in PBS with 0.1% Tween-20 (PBST) and incubated for 1 h at room temperature. Immunoblotting was performed by incubating the membranes in 5% milk-PBST overnight at 4°C with rabbit monoclonal anti-PTEN (Santa Cruz, USA) at 1∶1000. The membranes were washed three times in PBST, and incubated for 1 h with horseradish peroxidase (HRP)-conjugated secondary antibody (Zhongshan, Beijing, China). After washing three times with PBST, protein bands were developed using enhanced chemiluminescence reagents (Beyotime) and quantified by densitometry with Quantity One version 4.4.0 software. PTEN expression was normalized to β-actin.

### EGFP Fluorescence Reporter Vector Analysis

Enhanced green fluorescent protein (EGFP) cDNA was removed from the pEGFP-N2 vector (Clontech) using restriction enzymes and subcloned into the pcDNA3.1 vector (Invitrogen). The 3′-UTR fragments from PTEN containing the predicted mmu-miR-141 binding site were cloned into pcDNA3.1/EGFP constructs at the BamHI and EcoRI sites. The mouse uterine stromal cells were cotransfected in 24-well plates with 0.5 µg of the EGFP report vector and 0.1 µg of the control vector containing pDsRed-C1. EGFP and RFP activities were measured using a fluorospectrophotometer. The primers used for the analysis are listed in Table 2.

**Table 2:** Sequences of primers.

Primer	Sequence (5′→3′)	
miR-141 PCR primer	Forward	CCGGGTAACACTGTCTGGTAAAG
	Reverse	GTGCAGGGTCCGAGGT
PTEN PCR primer	Forward	ACGGGATCCGTCTGTTAAACAGCCTTACTC
	Reverse	CAGGAATTCAATCAGTTTTAAGTGGAGTTT
	Mutation	CAGGAATTCAATCAGTTTTAAGTGGAGTTTATTTGTGAAAGAGATAAAGGGTTGTTAAC

### Injection

On D2, the pregnant mice were randomly divided into one of three groups: mimic, inhibitor, and control (15 mice per group). In mimic group, the left uteri horns were untreated and the right uterine horns were injected with mmu-miR-141 mimic. In inhibitor group, the left uteri horns were untreated and the right uterine horns were injected with mmu-miR-141 inhibitor. In control group, the left uteri horns were untreated and the right uterine horns were injected with mmu-miR-141 inhibitor empty plasmid. The mice were killed on D7. miR-141 and PTEN expression was detected to confirm the efficiency and specificity of in vivo interference by injection. The number of embryos at each uteri horns was recorded alone.

### H&E Staining

On D5, the whole mouse uterus was fixed in 4% paraformaldehyde, embedded in paraffin and 4-mm sections were cut. H&E staining was performed by Hematoxylin-Eosin Stain kit (Nanjing JianCheng Technology co., LTD)according to the manufacturer’s instructions.

### Bioinformatics analysis

Previous studies have carried out target gene prediction using several online tools, including miRGen [21], Targetscan [22], and Pictar [23].

### Statistical Analysis

Data were analyzed using SPSS statistical software (version 16.0; SPSS Inc., Illinois, USA). Values are represented as the means ± SD. Differences between groups were analyzed by Student’s t-test. For all statistical tests, differences were considered significant if P<0.05.

## Results

### Differential Expression of mmu-miR-141 in the Endometrium of Pregnant Mice

**Figure 1:**
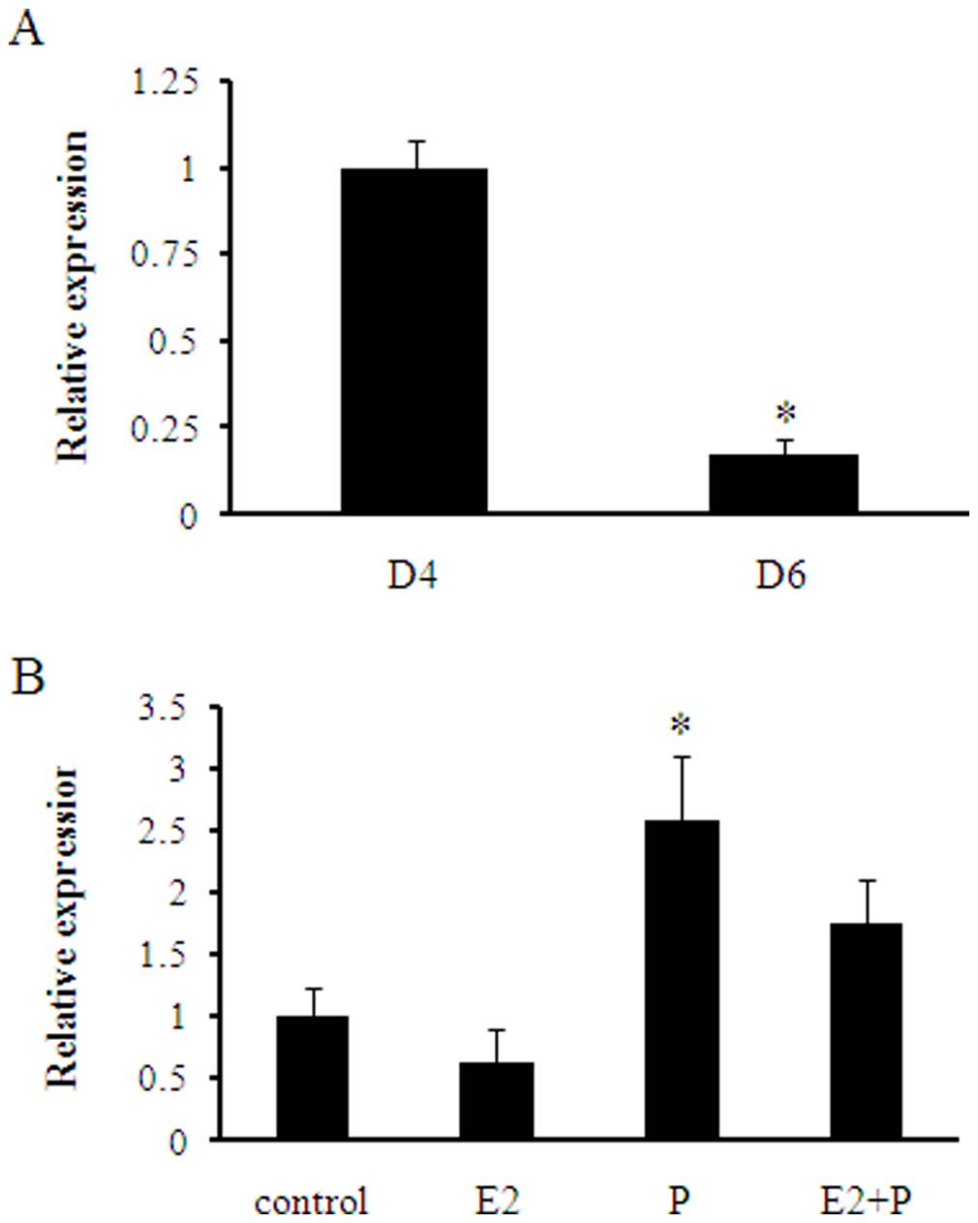
Real-time PCR analysis of mmu-miR-141 expression. The mmu-miR-141 expression level was significantly lower in D6 than in D4 (A). mmu-miR-141 expression in progesterone treated group was the most higher than the other groups (B). *P<0.05.

**Figure 2:**
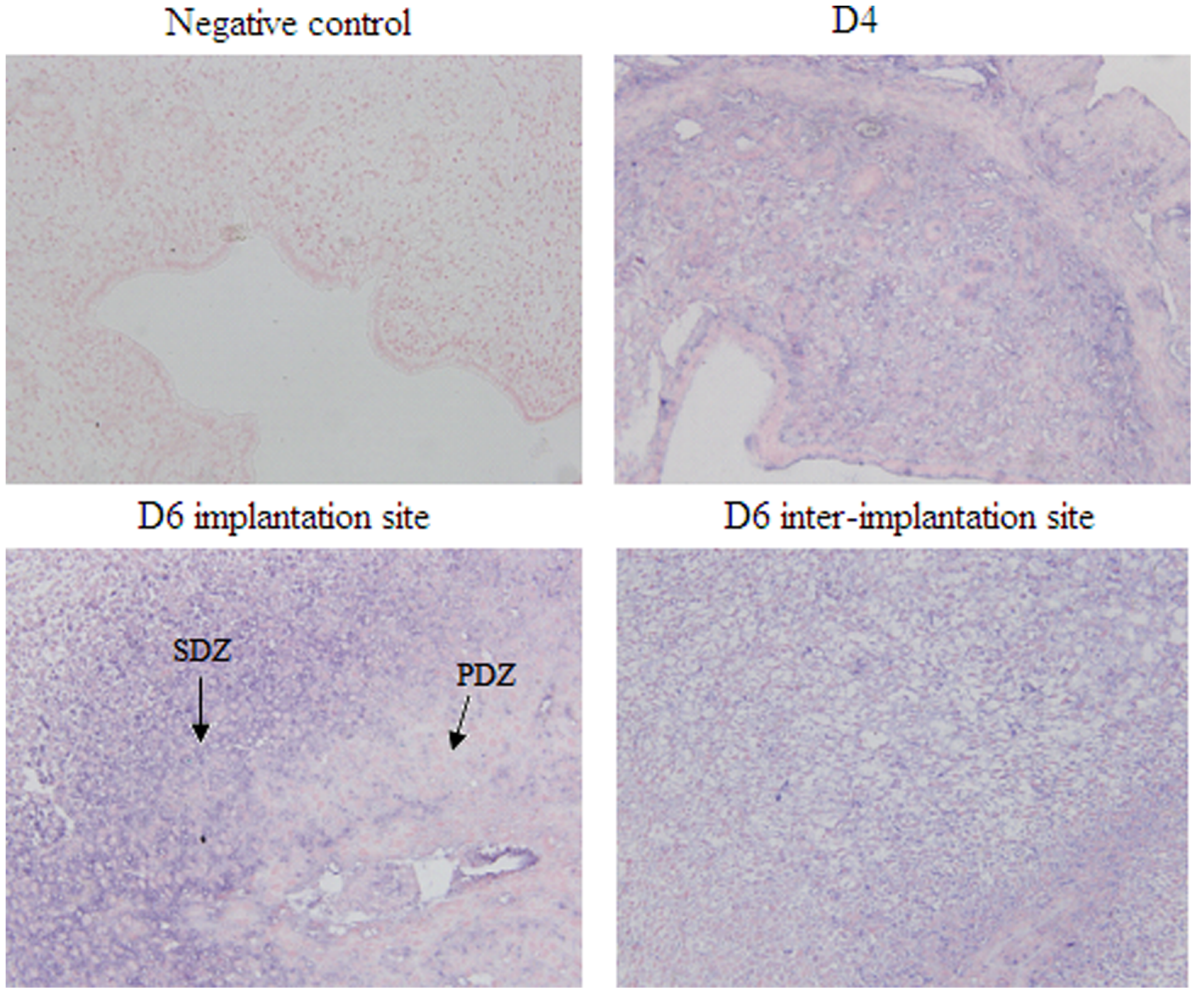
In-situ hybridization to determine mmu-miR-141 localization in the uterus during embryo implantation. mmu-miR-141 was primarily located in stromal cells. mmu-miR-141 expression was much higher in second decidual zone (SDZ) than that in primary decidual zone (PDZ) on D6 implantation sites. Bluish-violet staining was determined as positive. (×200).

In our previous study, miRNA chip analyses revealed that mmu-miR-141 expression in endometrial tissue was obviously lower after implantation (D6) than before implantation (D4). Here, real-time PCR was used to verify the mmu-miR-141 expression in mice uteri on D4 and D6. As shown in Figure 1A, mmu-miR-141 expression was significantly lower (fold change  = 0.1862, D6 vs. D4) on D6 than on D4 (P<0.05). These findings are consistent with the miRNA chip assay results (fold change  = 0.2575, D6 vs. D4). In addition, real-time PCR also showed that mmu-miR-141 expression in progesterone treated group was higher than the other groups (P<0.05, Figure 1B). The results of the in-situ hybridization showed that mmu-miR-141 was mainly located in the stromal cells. Moreover, mmu-miR-141 expression was much higher in second decidual zone (SDZ) than that in primary decidual zone (PDZ) on D6 implantation sites (Figure 2). The result suggested that mmu-miR-141 might involved with stromal cells proliferation.

### mmu-miR-141 Inhibitor Decreased Stromal Cells Proliferation

**Figure 3:**
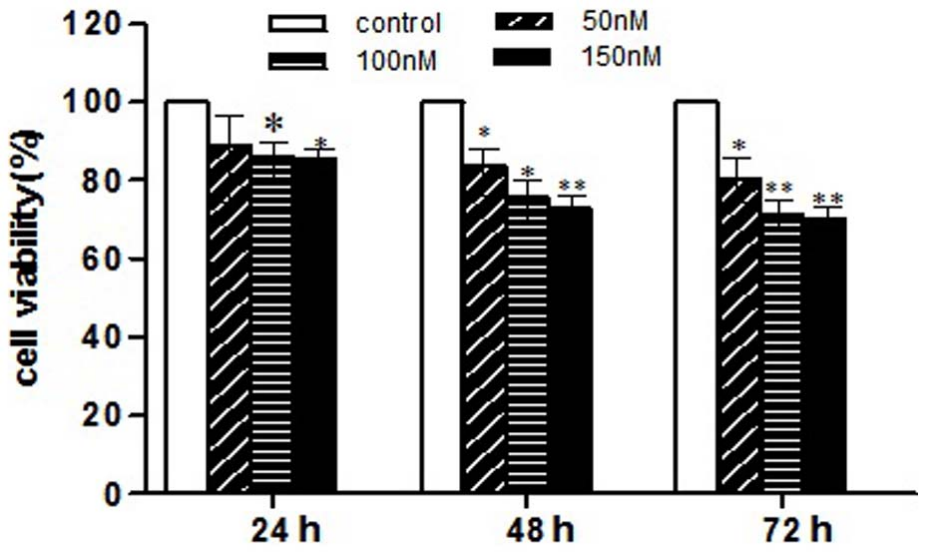
mmu-miR-141 inhibitor inhibited stromal cell growth. MTT assay revealed reduced cell viability in the mmu-miR-141 inhibitor-transfected stromal cells as compared to the control. This decrease in cell viability was observed at all concentrations of mmu-miR-141 (50, 100, and 150 nM) and at all incubation times (24, 48, and 72 h). *P<0.05, **P<0.01.

MTT assay was used to determine cell growth. The results revealed decreased cell growth in the mmu-miR-141 inhibitor-transfected stromal cells as compared to that in the control group (Figure 3). The difference in cell growth compared to the control was observed at all concentrations (50, 100, and 150 nM) and incubation times (24 h, 48 h, and 72 h) of mmu-miR-141. This result suggested that downregulation of mmu-miR-141 may inhibit cell proliferation.

### mmu-miR-141 Inhibitor Affected the Stromal Cell Cycle and Apoptosis

**Figure 4:**
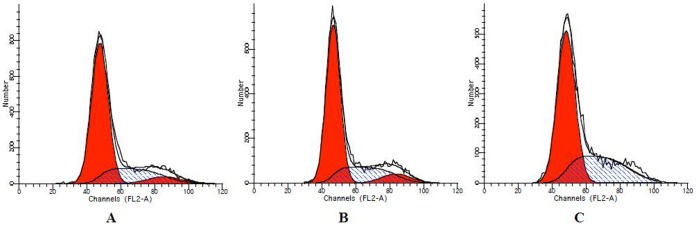
Effect of mmu-miR-141 inhibitor on cell cycle. Endometrial stromal cells transfected with mmu-miR-141 inhibitor exhibited S phase arrest, while G1 and G2 phases in these cells remained unaltered. (A) normal endometrial stromal cells. (B) negative control. (C) miR-141inhibitor.

**Figure 5:**
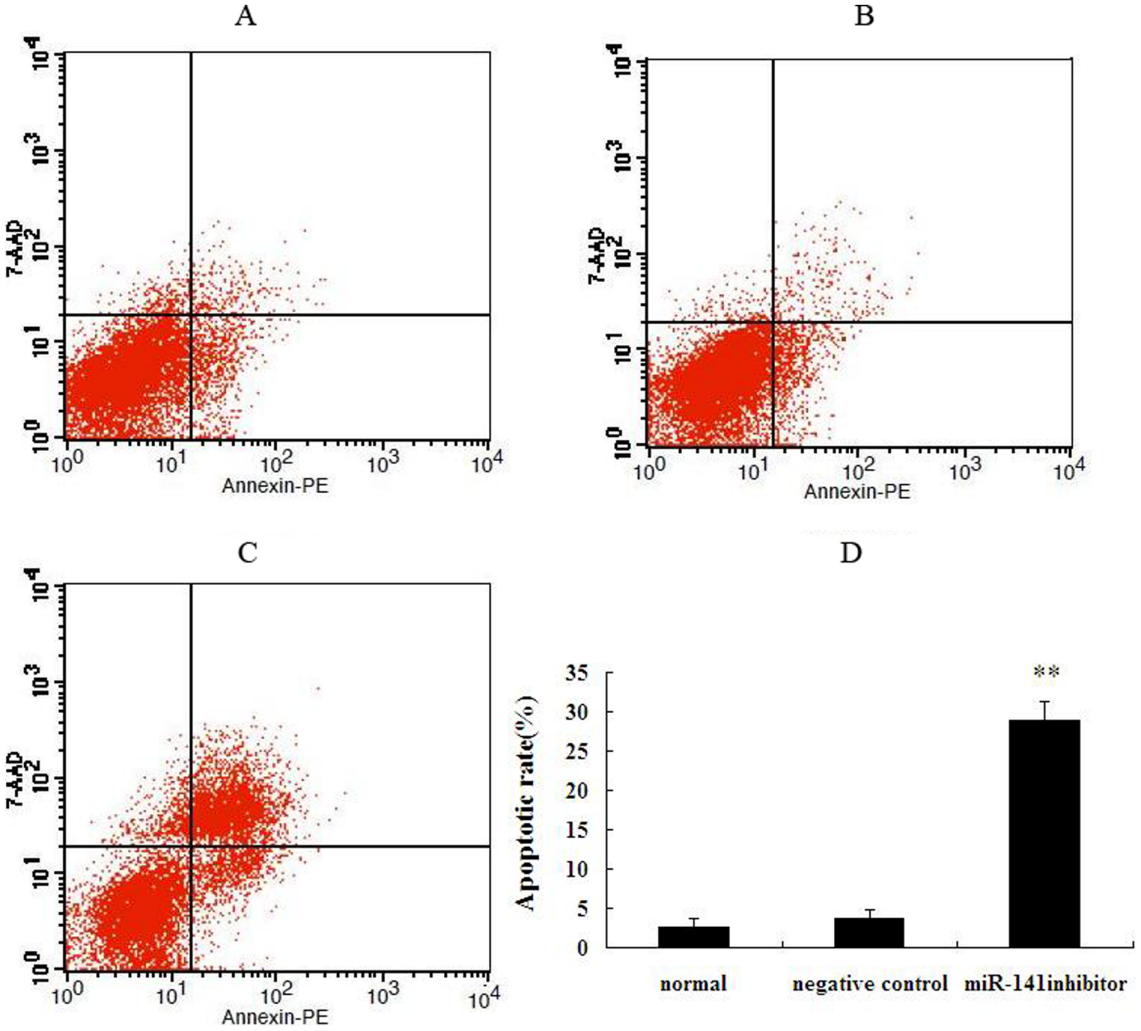
Effect of mmu-miR-141 inhibitor on apoptosis of endometrial stromal cells. Compared with normal endometrial stromal cells and negative control, the apoptotic rate of endometrium stromal cells transfected by mmu-miR-141 inhibitor was significantly increased. (A) normal endometrial stromal cells. (B) negative control. (C) miR-141 inhibitor. (D) quantification of the apoptotic rate. **p<0.01 as compared to the control.

The mmu-miR-141 inhibitor-transfected stromal cells were harvested for cell cycle and apoptosis analyses after culture for 72 h. Transfection of stromal cells with mmu-miR-141 inhibitor induced S phase arrest (G1 and G2 phases remained unaltered) and the apoptotic rate was significantly increased as compared to normal and negative control stromal cells (**P<0.01; Figures 4 and 5).

### mmu-miR-141 Regulated the PTEN Protein Expression in Endometrial Stromal Cells

We used bioinformatics analysis (miRGen, Targetscan, Pictar) to predict the potential target genes of mmu-miR-141. Results of the analysis revealed that PTEN, programmed cell death factor 4 (PDCD4), and *Kruppel*-like factor 6 (KLF6) were the potential target genes of mmu-miR-141, according to the function of the endometrium during embryo implantation. Bioinformatics analysis and investigating the degradation character of mmu-miR-141 revealed that PTEN, which plays a role in proliferation and apoptosis, was the most likely target gene of mmu-miR-141.

**Figure 6:**
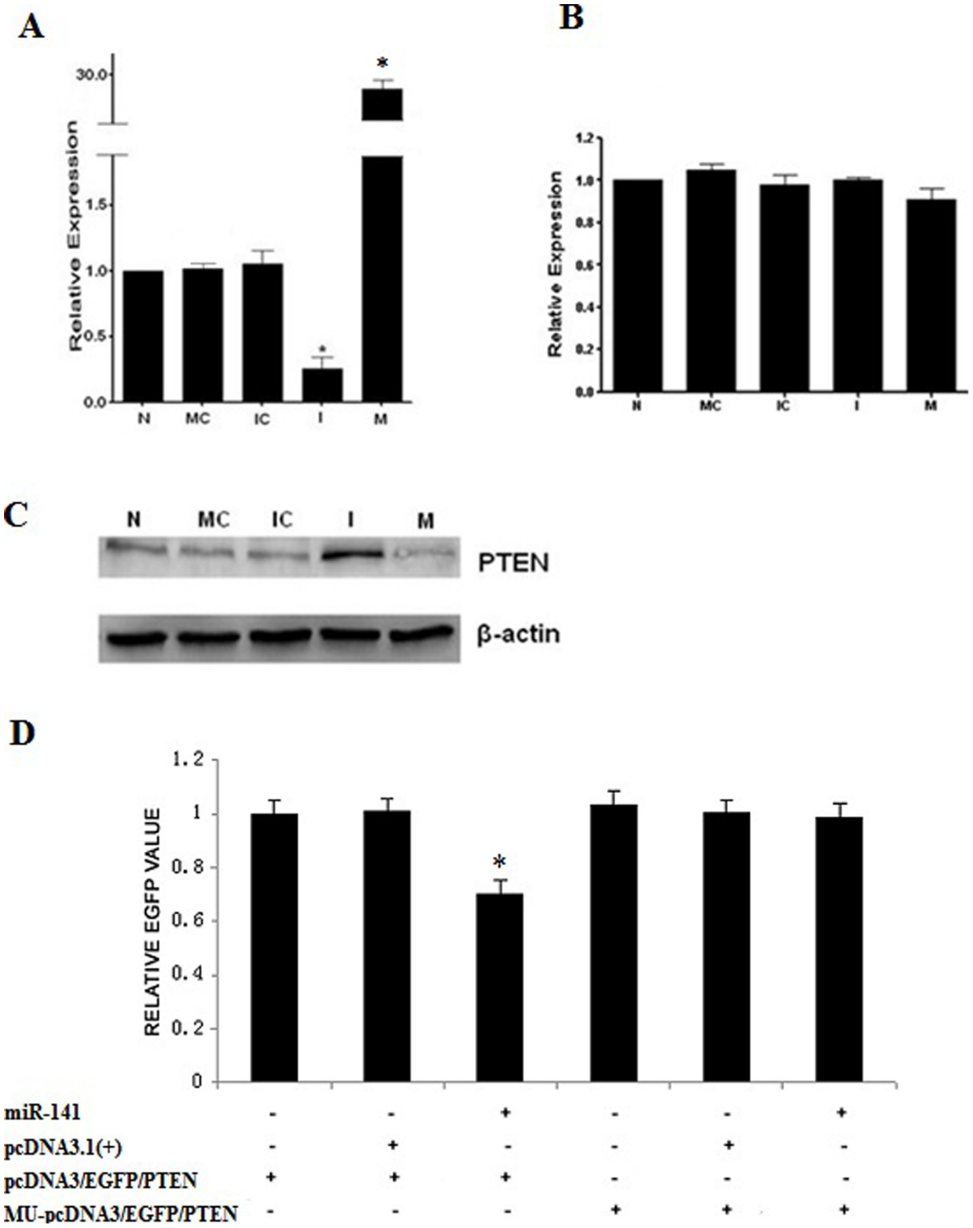
PTEN is a target gene of mmu-miR-141. Mmu-miR-141 expression (A) and PTEN mRNA level (B) were detected by real-time PCR in endometrial stromal cells transfected with mmu-miR-141 mimic or inhibitor. (C) PTEN protein level was detected by Western blot analysis. N: untreated cells. MC: control of mimic. IC: control of inhibitor. I: inhibitor. M: mimic. (D) EGFP fluorescence reporter vector analysis. The constructs MU-pcDNA3/EGFP/PTEN, pcDNA3/EGFP/PTEN, pcDNA3.1(+), and pcDNA3.1/pri-miR-141 were transfected into HEC-1B cells, and fluorescence was measured after 48 h. *P<0.05 compared with untreated cells.

To confirm that PTEN was indeed the target gene of mmu-miR-141, the mRNA and protein levels of PTEN were investigated after transfection of primary stromal cells with mmu-miR-141 mimic or inhibitor. As shown by the results of the real-time PCR (Figure 6A and B), the mRNA levels of PTEN did not change in the cells transfected with the mmu-miR-141 mimic or inhibitor. However, results of Western blotting showed that the protein level of inhibitor-treated cells was significantly increased, while it was obviously decreased in the mimic-treated cells (Figure 6C). Furthermore, results of the EGFP fluorescence reporter vector analysis indicated that the EGFP expression level was the lowest in pcDNA3/EGFP/PTEN and miR-141-transfected cells, indicating that miR-141 significantly inhibited EGFP expression as compared to the control (P<0.05), whereas the EGFP expression remained unaffected in the MU-pcDNA3/EGFP/PTEN-transfected cells (Figure 6D). This result confirms that miR-141 targets the 3′-UTR of the PTEN mRNA in stromal cells. All these results suggested that mmu-miR-141 regulates protein expression of PTEN in stromal cells and that PTEN is a target gene of mmu-miR-141.

### Injection with mmu-miR-141 Mimic and Inhibitor Decreased Implantation Rate

**Figure 7:**
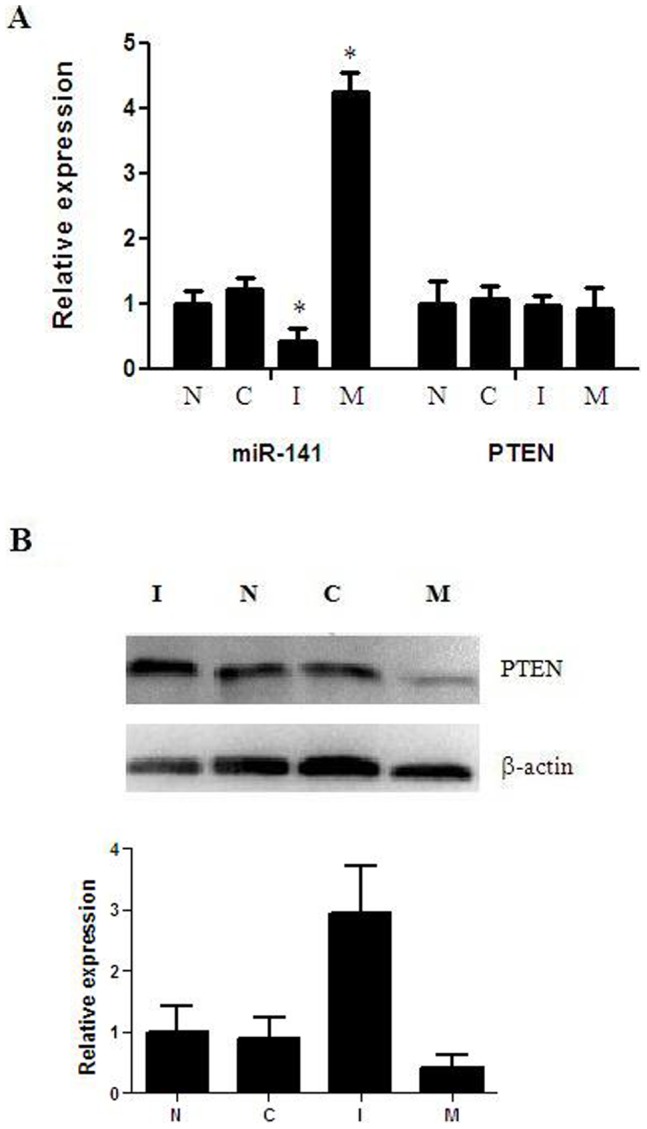
Expression of mmu-miR-141 and PTEN after injection. (A) mRNA levels of mmu-miR-141 and PTEN by Real-time PCR. (B) Protein levels of PTEN by western blot. *P<0.05 compared with untreated uteri horns.

**Figure 8:**
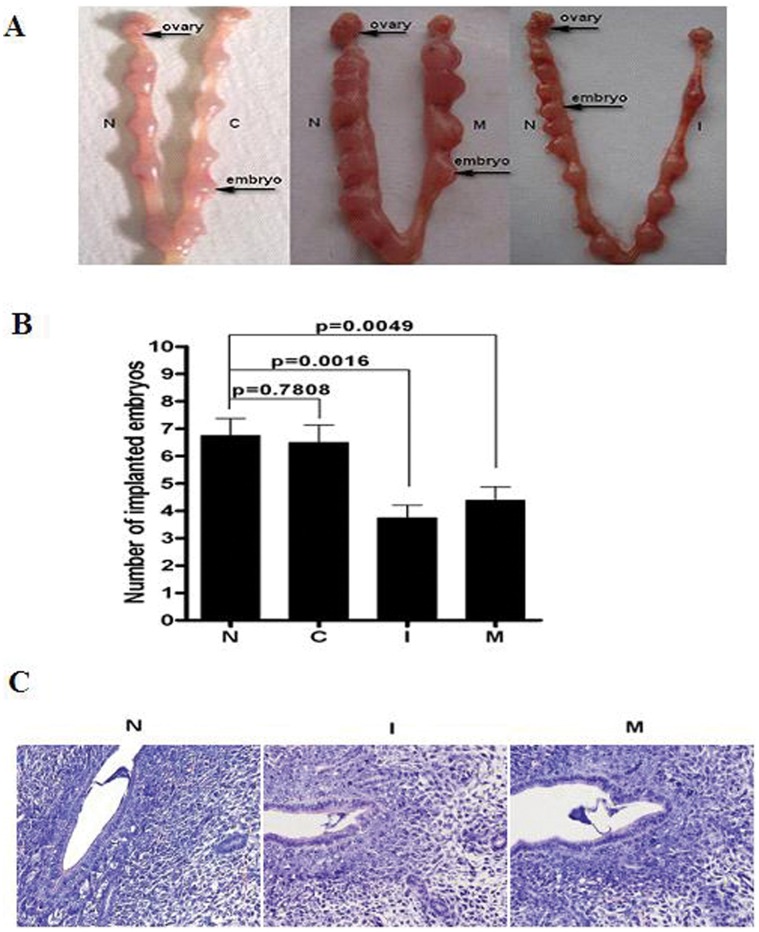
Effect of mmu-miR-141 inhibitor or mimic on embryo implantation. The number of embryos in the mimic (P  = 0.0049) and inhibitor (P  = 0.0016) treated uteri horns were significantly lower than that in the untreated uterine horn(A, B). H&E staining was used to show blastocyst attachment to the luminal epithelium at implantation sites on D5 and no obviously changes was observed in inhibitor and mimic treated uteri horns at implanted sites respectivity(C)(×400). N: untreated uterine horn. C: control(empty plasmid). I: treated with mmu-miR-141 inhibitor. M: treated with mmu-miR-141 mimic.

Compared with untreated uteri horns, miR-141 was obviously decreased in inhibitor treated uteri horns, while obviously increased in mimic treated uteri horns. PTEN mRNA expression has no difference among the four treatment. However, PTEN protein was significiantly increased in inhibitor treated uteri horns and obviously decreased in mimic treated uteri horns (Figure 7). The number of embryos in all the groups was recorded on D7. As shown in Figure 8A and B, there were 6.75±0.6196, 6.50±0.6268, 3.75±0.4532, and 4.37±0.4978 embryos in the untreated, control, inhibitor, and mimic treated uteri horns respectivity. There were no significant differences between the untreated and control uteri horns (P  = 0.7808). However, there were significant differences in the number of embryos in the mimic (P  = 0.0049) and inhibitor (P  = 0.0016) uteri horns as compared with the untreated uteri horns. In addition, to verify whether the decreased number of implanted embryo on D7 was due to failure implantation, H&E staining was used to show blastocyst attachment to the luminal epithelium at implantation sites on D5 and we did not observe obviously changes in inhibitor and mimic treated uteri horns at implanted sites respectivity (Figure 8C). It suggested that the process after blastocyst attached to the luminal epithelium was abnormal. It needs to be studied in further.

## Discussion

MiR-141 is a member of the miRNA-200 family that is considered to function as an oncogene by promoting cell proliferation and inhibiting cell apoptosis. A previous study has confirmed that silencing of miR-141 would completely inhibit the proliferation of JEG-3 cells [18]. In another study, Imanaka et al. found that miR-141 conferred resistance to cisplatin-induced apoptosis by directly targeting the 3′-UTR of YAP1 in human esophageal squamous cell carcinoma [24]. Alterations in the miR-141 expression have also been shown to affect tumor growth and response to chemotherapy. MiR-141 was highly expressed in prostate cancer, endometriosis, and ovarian carcinoma [25]–[27]; it is also associated with the prognosis of serous ovarian carcinoma [27].

Based on the results of the miRNA chip assay in our previous study, we studied in detail the possible role of miR-141 during embryo implantation in this research. We observed that the expression of miR-141, which was mainly located in stromal cells, was higher on the day before implantation (D4) than after implantation (D6). In addition, mmu-miR-141 expression was much higher in SDZ than that in PDZ on D6 implantation site. According to the proliferation pattern of endometrial cells, epithelial cells stop to proliferation and begin to differentiation on D4, stromal cells in PDZ stop to proliferation on D6, but stromal cells in SDZ start to proliferation [28]. Therefore, we speculated that mmu-miR-141 might take part in the proliferation of stromal cells. At the time of implantation, the uterine endometrium responds to blastocyst implantation with extensive proliferation and differentiation of stromal cells into decidual cells, which then undergo apoptosis [29]. The balance between cell proliferation and apoptotic cell death is crucial for successful embryo implantation and the maintenance of pregnancy [29].

Inhibitor-transfected stromal cells were harvested on D4 for cell cycle and apoptosis analysis. Downregulation of miR-141 was found to inhibit cellular proliferation and promote apoptosis by inducing S phase arrest. This confirmed that the differential expression of miR-141 before and after implantation was associated with cellular proliferation and apoptosis by which stromal cells differentiated into decidual cells.

Results of the bioinformatics analysis indicated that PTEN, PDCD4, and KLF6 were the potential target genes of mmu-miR-141. According to some studies, the deletion and mutation of KLF6 and PDCD4 were associated with gastric cancer and colon carcinogenesis, since the downregulation of these cancer suppressor genes were found to promote over-proliferation of cells [30], [31]. Furthermore, Akt phosphorylation was accompanied by the loss of PTEN in clinical specimens of endometrial carcinomas [32], [33]. PTEN1 was frequently mutated and delete in primary endometrial carcinomas [34]. Researchers also found that miR-141 expression was higher in adenocarcinoma tissues than in paracancerous tissue in endometrioid adenocarcinoma [35]. Chen et al. found that PTEN expression was higher in pregnant mice than in non-pregnant mice, with a steady increase from D1 to D7 and reaching the maximal level on D5 of pregnancy, and the number of implantation sites was significantly decreased after PTEN antisense oligonucleotides were injected in the mice on D3 [36]. In our study, we found that PTEN protein expression was inhibited by the upregulation of mmu-miR-141 and increased by its downregulation, whereas the mRNA level remained unaltered regardless of the status of mmu-miR-141. EGFP fluorescence reporter vector analysis also confirmed that miR-141 targets the 3′-UTR of the PTEN mRNA in stromal cells. Along with the results of previous studies and our study results, we assumed that PTEN is the target gene of mmu-miR-141.

In our study we also found that after the physiological mmu-miR-141 level was altered on D2 by injecting with inhibitor or mimic. After injected with inhibitor or mimic of miR-141, although blastocyst could attach to the luminal epithelium on D5, the embryo implantation sites were significantly decreased on D7. It suggested that the process after blastocyst attached to the luminal epithelium was abnormal. miR-141 has been verified to promote cell proliferation and inhibit cell apoptosis in many cancers and in the miR-141 inhibitor-transfected stromal cells, we also observed the decreased proliferation and increase apoptosis. Furthermore, PTEN, the target gene of mmu-miR-141, has been verified to inhibit cell proliferation and induce apoptosis by its downstream moleculars such as Akt, IGF-IR [37], [38]. As we known, the balance between proliferation and apoptosis of endometrium was crucial for embryo implantation. After injected with inhibitor or mimic of miR-141, the stromal cells proliferation and apoptosis were both unusual and the balance between proliferation and apoptosis of endometrium would be destroyed which make against embryo implantation normally. So we speculated that the altered miR-141 level may induce blastocyst invaded into endometria abnormally after attached to the luminal epithelium. All the results indicated that mmu-miR-141 may influence cellular proliferation and apoptosis in the endometrium by negatively regulating PTEN expression and consequently play an essential role in embryo implantation. The mechanism of action of mmu-miR-141 should be investigated in greater detail in future studies.
